# A Multi-Species Investigation of Sponges’ Filtering Activity towards Marine Microalgae

**DOI:** 10.3390/md20010024

**Published:** 2021-12-24

**Authors:** Despoina Varamogianni-Mamatsi, Thekla I. Anastasiou, Emmanouela Vernadou, Nikos Papandroulakis, Nicolas Kalogerakis, Thanos Dailianis, Manolis Mandalakis

**Affiliations:** 1 Institute of Marine Biology, Biotechnology & Aquaculture, Hellenic Centre for Marine Research, 715 00 Heraklion, Greece; d.varamogianni@hcmr.gr (D.V.-M.); theanast@hcmr.gr (T.I.A.); e.vernadou@hcmr.gr (E.V.); npap@hcmr.gr (N.P.); 2 School of Chemical and Environmental Engineering, Technical University of Crete, 731 00 Chania, Greece; nicolas.kalogerakis@enveng.tuc.gr

**Keywords:** Mediterranean sponges, microalgae, filtering capacity, bioremediation, integrated aquaculture, cell retention

## Abstract

Chronic discharge of surplus organic matter is a typical side effect of fish aquaculture, occasionally leading to coastal eutrophication and excessive phytoplankton growth. Owing to their innate filter-feeding capacity, marine sponges could mitigate environmental impact under integrated multitrophic aquaculture (IMTA) scenarios. Herein, we investigated the clearance capacity of four ubiquitous Mediterranean sponges (*Agelas oroides*, *Axinella cannabina*, *Chondrosia reniformis* and *Sarcotragus foetidus*) against three microalgal substrates with different size/motility characteristics: the nanophytoplankton *Nannochloropsis* sp. (~3.2 μm, nonmotile) and *Isochrysis* sp. (~3.8 μm, motile), as well as the diatom *Phaeodactylum tricornutum* (~21.7 μm, nonmotile). In vitro cleaning experiments were conducted using sponge explants in 1 L of natural seawater and applying different microalgal cell concentrations under light/dark conditions. The investigated sponges exhibited a wide range of retention efficiencies for the different phytoplankton cells, with the lowest average values found for *A. cannabina* (37%) and the highest for *A. oroides* (70%). The latter could filter up to 14.1 mL seawater per hour and gram of sponge wet weight, by retaining 100% of *Isochrysis* at a density of 10^5^ cells mL^−1^, under darkness. Our results highlight differences in filtering capacity among sponge species and preferences for microalgal substrates with distinct size and motility traits.

## 1. Introduction

With the gradual increase of global population, and consequently of fish food demand, the aquaculture industry has rapidly expanded over the last few decades [1]. As a result, high organic and nutrient loadings, generated from aquaculture activities (e.g., feed wastage, fish excretion, and fecal production), are continuously being released into the seawater [2], occasionally causing detrimental effects in the surrounding environment, such as toxic algal blooms, eutrophication and anoxia [3]. Such effects are particularly likely at aquaculture settings located in sheltered areas such as gulfs and bays [4]. 

The Integrated Multi-Trophic Aquaculture (IMTA) concept is a newly introduced approach, firstly developed in Asia [5], in which the by-products from one species are recycled to become input for another, thus minimizing the environmental impact caused by intensive aquaculture practices. These systems include the aquaculture unit of a main farmed species (e.g., finfish) in proximity with the rearing of secondary species, typically belonging to lower trophic levels, such as filter-feeders [6]. 

Sponges (Porifera), the oldest extant filter-feeding macroinvertebrates [7], have recently been viewed as promising candidates for IMTA scenarios [8,9] due to their capability to filter large volumes of water [10,11,12] and retain microorganisms or other particles of various sizes, ranging from 0.1 to 50 μm [13,14,15], with high efficiency (75–99%) [11,14,16,17]. Having developed intricate patterns of symbiotic associations with microbial communities, sponges are among the most diverse and complex holobionts in the marine environment [18] and possess unique feeding mechanisms. Associated microbiomes render sponges capable of distinct nutritional strategies that extend beyond the standard heterotrophy described above. Hence, depending on the species and environmental parameters, sponges can benefit from photosynthesis [17], or feed on dissolved organic matter (DOM) [19]. In addition to these appealing characteristics, sponges do sustain a “gold mine” of bioactive compounds with pharmaceutical [20] and cosmetic potential [21], while the biomass of some species can be exploited for the production of bath sponges [22]. By offering several valorization opportunities, cultivation of sponges can become an extra source of profit for fish farmers and, thus, their inclusion in IMTA systems is rather tempting. 

The first study investigating microalgae as test particles in sponge filtering experiments was conducted by Frost [23]. Therein, the clearance effect of the freshwater sponge *Spongilla lacustris* was examined upon the unicellular green microalga *Chlamydomonas reinhardtii*, among other microbial species, and it was found to be capable of filtering up to 0.055 mL of water per second and per gram of sponge wet weight. In a later study, Riisgård et al. (1993) [24] related the filtering activity and pumping energy cost of the marine sponge *Halichondria panicea* with temperature, after experimenting with flagellated cells of the microalgal species *Rhodomonas* sp. as test particles. By using flow cytometry, Pile and Witman (1996) [14] investigated in situ feeding of the boreal sponge *Mycale lingua* on heterotrophic and autotrophic plankton and found that 86% of the autotrophic eucaryotes of 3 to 10 μm, can be efficiently retained. Similarly, Ribes et al. (1999) [15] studied in situ the natural diet of the marine sponge *Dysidea avara* (Schmidt) throughout an annual cycle, and they concluded that microalgae constitute a significant percentage of the marine sponge diet, with pico- and nanoeucaryotes contributing 11 ± 3% and larger phytoplankton accounting for 11 ± 10%. An interesting finding was also reported by Osinga et al. (2001) [25], who tested the tropical sponge *Pseudosuberites* (aff.) *andrewsi* with a wide range of microalgal cell concentrations of the marine species *Dunaliella tertiolecta* (5–8 μm) under laboratory conditions. It was demonstrated that the sponge filtration rate dropped dramatically at concentrations higher than approximately 4 × 10^5^ cells mL^−1^. 

However, none of these cases conceptualized marine sponges as living bioremediation agents and specifically aimed to compare the filtering power of various species for the reduction of phytoplanktonic biomass near aquaculture facilities. Until now, the majority of research was focused on the removal of bacteria from seawater [8,9,26,27,28]. In this study, we investigate the inherent filtering capacity and selectivity of four Mediterranean marine demosponges thriving in Greek waters, namely *Agelas oroides*, *Axinella cannabina*, *Chondrosia reniformis* and *Sarcotragus foetidus*, against three representative marine microalgae species of different size and motility characteristics: *Nannochloropsis* sp., *Isochrysis* sp. and *Phaeodactylum tricornutum.* In addition, we assess the reproducibility of sponges’ cleaning performance, and we examine how the initial cell concentration in seawater and light intensity can affect filtering capacity. Our results provide valuable insights into the suitability of sponge species as bioremediators in IMTA systems, or other impacted environments with high microalgae loading. To the best of our knowledge, this is the first report to systematically assess filtering activity over a broad range of sponges, microalgal substrates and different experimental setups. 

## 2. Results and Discussion

### 2.1. Assessement of Reproducibility

**Figure 1:**
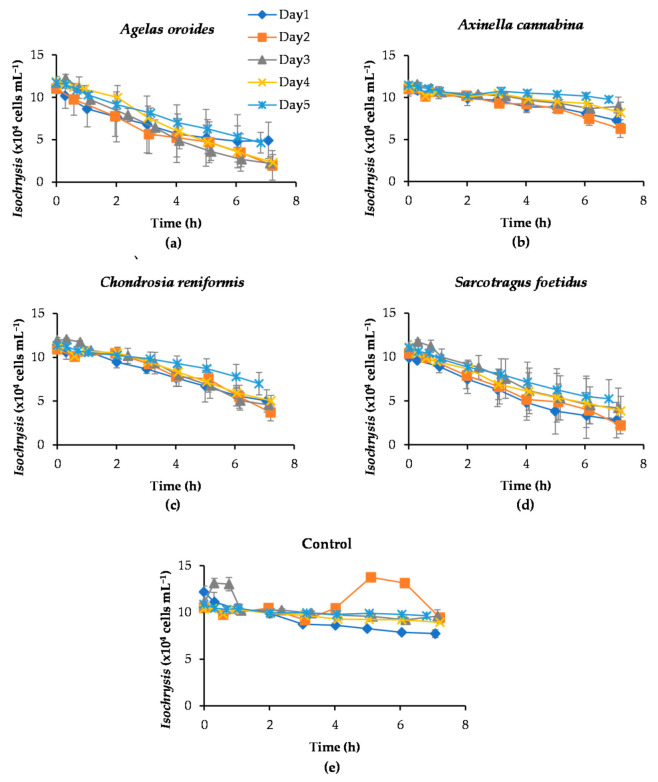
Removal of the marine microalga *Isochrysis* by the four study species of sponges over five consecutive days. The 7-h decrease of *Isochrysis* by (**a**) *Agelas oroides*, (**b**) *Axinella cannabina*, (**c**) *Chondrosia reniformis*, (**d**) *Sarcotragus foetidus* and (**e**) control samples (without sponge explants) are presented. In each experiment, the initial concentration of *Isochrysis* was set at 10^5^ cells mL^−1^. The error bars represent the standard deviation obtained from the five biological replicates of each sponge species.

The depletion of *Isochrysis* cells by the four sponge species *Agelas oroides*, *Axinella cannabina*, *Chondrosia reniformis* and *Sarcotragus foetidus* over the series of five consecutive experiments conducted for seven hours each is shown in Figure 1. The initial concentration of *Isochrysis* was 10^5^ cells mL^−1^ and presented results correspond to the average depletion derived for each species using data from five biological replicates. The average wet weight of sponge fragments was 71.6 ± 6.7 g for *Agelas oroides*, 55.5 ± 4.7 g for *Axinella cannabina*, 82.6 ± 6.2 g for *Chondrosia reniformis* and 113.3 ± 29.1 g for *Sarcotragus foetidus*. These values were assumed to remain constant throughout the experiments.

**Figure 2:**
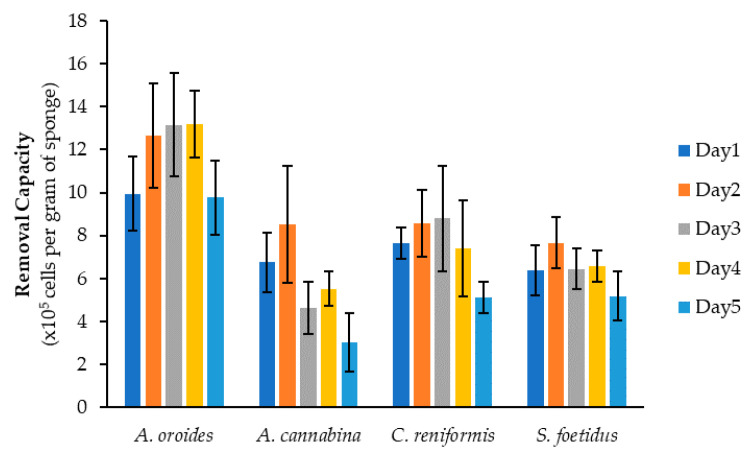
Average retention of *Isochrysis* cells by *Agelas oroides*, *Axinella cannabina*, *Chondrosia reniformis* and *Sarcotragus foetidus* after 7 h of exposure and over five consecutive experiments. Error bars indicate the standard deviation obtained from the five biological replicates of each sponge species.

Microalgae concentration in natural seawater (NSW) decreased markedly within the tested seven hours, following Coughlan’s exponential model [29]. This depletion was clearly the result of sponges’ filtering activity and not of other causes, such as cell settling or lysis, as the concentration of microalgae in the control group remained rather constant over time (Figure 1e). The pattern of results obtained from each sponge species over the five consecutive experiments demonstrated high similarity, implying a rather stable filtration performance. However, it is worth noticing that the consumption of microalgal cells on the last day of the experiments (Day 5) tended to be lower for all sponges (Figure 1 and Figure 2). It is likely that sponge’s aquiferous system experienced partial saturation effects (e.g., clogging) as a result of continuous and excessive intake of microalgae, which eventually led to reduced pumping activity [25]. The discrepancy observed for *C. reniformis* was more pronounced, but still of marginal importance. Even in this case, the concentration decrease of microalgae measured over the course of Day 5 deviated only 14% from those detected during the previous four days.

Another interesting aspect of these preliminary experiments was the considerable biological variation (i.e., among different explants) in the results of the species *A. oroides* and *S. foetidus* throughout the 7-h experiment, as illustrated by the large error bars in Figure 1a,d. This is probably the result of weight differences between sponge fragments or of physiological variations (i.e., different number of oscules among explants). Nevertheless, this finding comes as no surprise, given the extensive within-sponge variances that have also been reported in other studies (e.g., Frost [23]).

Figure 2 demonstrates the removal capacity of the examined sponge species against *Isochrysis* cells after 7-h of exposure and over five consecutive days. In general, all sponges were able to efficiently retain significant quantities of microalgal cells, with a daily average exceeding 5 × 10^5^ cells per gram of sponge wet weight. The performance of all species demonstrated some day-to-day fluctuation, but *A. oroides* was clearly the most efficient filter-feeder, as it could retain the highest amounts of *Isochrysis* cells on all days (up to 10^6^ cells per gram wet weight). The highest cell removal capacity achieved by this species was 1.3 × 10^6^ cells g^−1^ and it was observed on Days 3 and 4. During the second experiment, the capacity of *A. oroides* was slightly reduced, while the lowest values were observed during the first and the fifth day. Concerning *C. reniformis*, the second most efficient sponge with an average removal capacity of 7.5 ± 1.5 × 10^5^ cells g^−1^, its performance maximized on Days 2 and 3 (8.6 and 8.8 × 10^5^ cells g^−1^, respectively). Slightly lower performance was evident on Days 1 and 4 (7.6 and 7.4 × 10^5^ cells g^−1^, respectively), and this was further reduced on Day 5 (5.1 × 10^5^ cells g^−1^). A similar day-to-day variability was also observed for *S. foetidus*, which exhibited an average removal capacity of 6.5 ± 0.9 × 10^5^ cells g^−1^. Among the four study species, *A. cannaniba* demonstrated the lowest removal cap acity with an average daily value of 5.7 ± 2.1 × 10^5^ cells g^−1^ and the most striking day-to-day fluctuation. Indeed, the capacity of this species on Day 2 (8.5 × 10^5^ cells g^−1^) decreased almost 3-fold on Day 5 (3.0 × 10^5^ cells g^−1^).

Over the first four days, the variance in the removal capacity of all sponge species was of no statistical significance (*A. oroides*: *p* = 0.16, *A. cannabina*: *p* = 0.06, *C. reniformis*: *p* = 0.19, *S. foetidus*: *p* = 0.46). Despite the compromised performance observed for all sponges during Day 5, one-way ANOVA revealed that the variation during the entire 5-day experimental period was significant only for the species *A. cannabina* (*p* = 0.004) and *C. reniformis* (*p* = 0.0005). Although a previous in situ study reported that sponges’ filtering activity can vary substantially on a time scale of a few days [11], our lab-based investigation showed that all examined species were capable of maintaining their filtering performance for at least four or five consecutive days of 7-h exposure to microalgae. The fairly stable performance of sponges might be indicative of their successful adaptation to tank conditions and their healthy physiological status, while it could be also attributed to the highly-controlled experimental conditions. 

**Figure 3:**
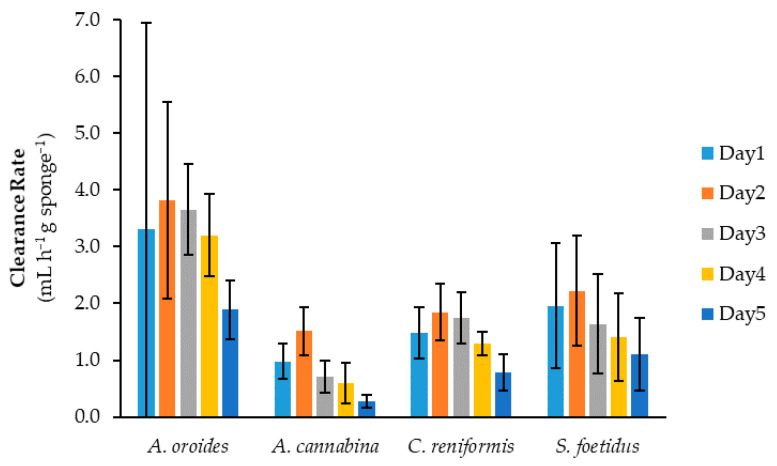
Calculated clearance rates of *Agelas oroides*, *Axinella cannabina*, *Chondrosia reniformis* and *Sarcotragus foetidus* for a fixed initial concentration of *Isochrysis* (10^5^ cells mL^−1^) over five consecutive experimental days. Error bars indicate the standard deviation obtained from the five biological replicates of each sponge species.

In Figure 3, the clearance rates (*c*) of the four sponge species derived for *Isochrysis* cells using Equation (1) are presented for the five experimental days. Similarly with the removal capacity (Figure 2), *A. oroides* showed the highest clearance rates, as it was capable of cleaning almost 4 mL of NSW per hour and per gram of sponge wet weight, while the lowest values were found for *A. cannabina* species (<1 mL h^−1^ g^−1^). Most species exhibited the highest clearance rates on Day 2, followed by a gradual decrease thereafter. Once again, the lowest values were observed on Day 5 for all species. The variance of clearance rate values over the 5-day experimental period was significant only for *A. cannabina* (*p* < 0.0001) and *C. reniformis* (*p* = 0.003), while no significant variation was found for *A. oroides* and *S. foetidus* (*p* = 0.52 and *p* = 0.33, respectively).

It should be also noted that the daily *c* values of some species (e.g., *A. oroides* and *S. foetidus*) were accompanied by high biological variation. This is not surprising as similar findings have been reported in previous studies [8,23,26]. In general, the comparison of clearance rates between different species, and particularly those obtained from different studies, is a tricky task due to the inherent variability of each species, the dissimilarities in the size of sponge used and their morpho-physiological features, the different types of microalgae tested, as well as the diverse units in which *c* are expressed [15,30]. Nevertheless, the clearance rates that we measured for the four sponge species were consistently lower than the rate reported by Frost [23] for the freshwater sponge *Spongilla lacustris* (198 mL h^−1^ g^−1^), which was tested with the microalga *C. reinhardtii* (6.6 μm) at the same initial cell concentration. A possible explanation could be that *S. lacustris* is a freshwater sponge dwelling in lakes, where eutrophication events and high concentrations of microalgae are much more common than in open-sea systems, especially the oligotrophic ones at the Eastern Mediterranean [31]. In addition, the microalga used in that study was almost twice the size of the *Isochrysis* cells used in our experiments. Furthermore, much higher rates have also been observed by Turon et al. [32], who tested *Dysidea avara* (426 mL h^−1^ g^−1^) and *Crambe crambe* (432 mL h^−1^ g^−1^) with latex spheres of the same size (4 μm) and at a similar initial concentration as in our experiments. However, these rates were derived using the dry weight of sponges, while it is questionable whether microalgae retention can be approximated by latex beads.

Another possible explanation for this apparent discrepancy could be the significantly larger sponge fragments used in our experiments, compared to those tested by Frost (0.97 to 2.22 g) [23] and Turon [32] (approximately 0.63 g for *D. avara* and 0.49 g for *C. crambe*). Decreasing clearance rates are typically observed for sponges of increasing size and this is mainly due to the lower number of living choanocytes present per unit weight in larger sponges. This trend was acclaimed by Ribes et al. [15], who additionally argued that the use of larger sponges may lead to water refiltration in the experimental chamber and cause a shift of rates towards lower values.

### 2.2. Effect of Cell Size on Cleaning Capacity

Several studies have shown that sponges are either capable [15,32,33] or incapable [14,23,34] of discriminating food particles based on their size. While this issue is not entirely clear, sponges are indeed suggested to use different mechanisms for selectively feeding on bigger or smaller particles [35]. In particular, particles with size smaller than approximately 5 μm are captured by the choanocytes, the flagellated cells of choanoderm, which are also responsible for the creation of sponges’ water current. The larger particles are primarily ingested through phagocytosis by pinacocytes, which line the incurrent canals [11,36,37].

**Figure 4:**
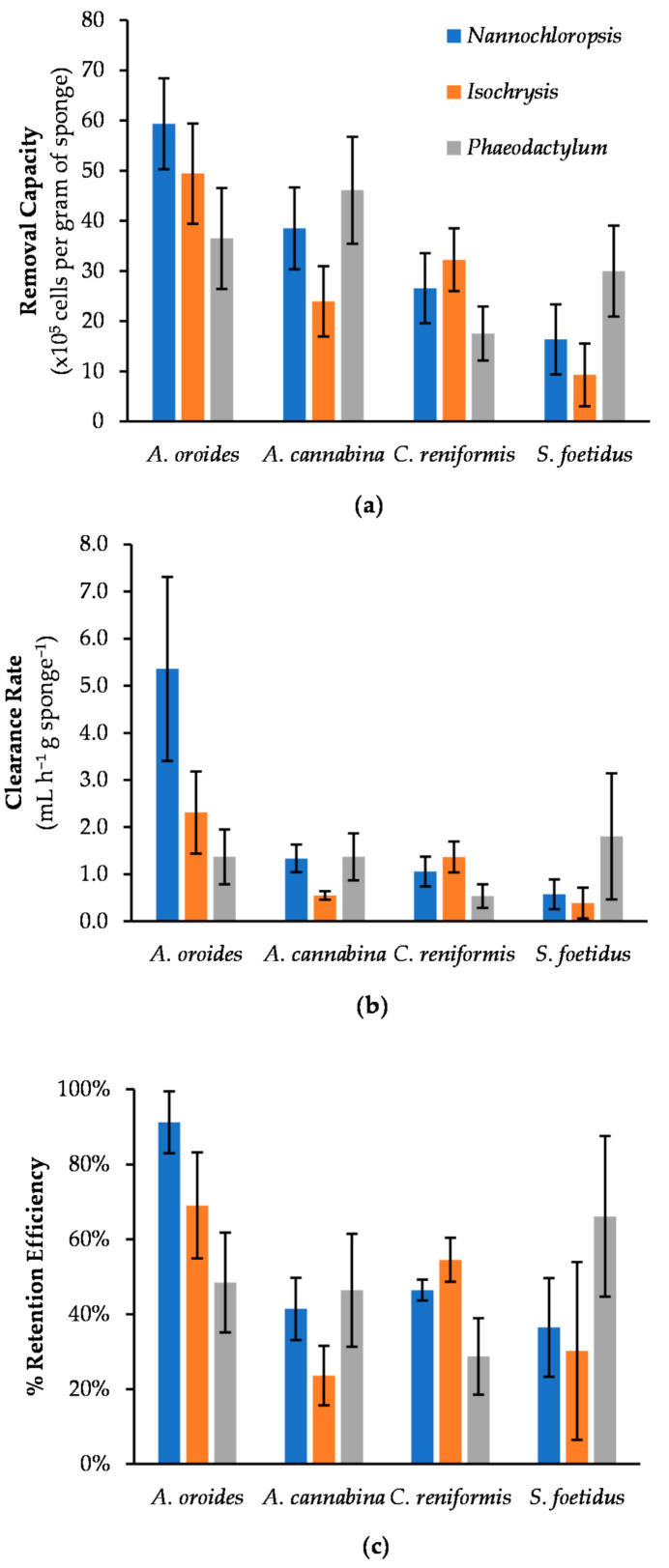
(**a**) Cell removal capacity, (**b**) clearance rate and (**c**) retention efficiency of sponges *A. oroides*, *A. cannabina*, *C. reniformis* and *S. foetidus* for three different types of marine microalgae prepared at the same initial concentration (5 × 10^5^ cells mL^−1^).

In our study, we tested two nanophytoplankton with minimal size differences and a diatom of much larger size as feed particles for sponges, all suspended at the same initial concentration of 5 × 10^5^ cells mL^−1^. Figure 4 presents a comparison regarding the filtering performance of the four study sponge species against the different types of cells. All performance metrics suggested that the investigated sponges have different food preferences. With regard to clearance rates, Coughlan’s model demonstrated a perfect fit to the time-series of microalgae concentration data, with the coefficient of determination being higher than 0.96 in most cases (Table 1). According to one-way ANOVA, each sponge presented statistically significant variance in its clearance rates among the three microalgae tested (*A. oroides*: *p* = 0.016; *A. cannabina: p* = 0.0008; *C. reniformis*: *p* = 0.0002; *S. foetidus*: *p* = 0.036).

The species *A. oroides* was more keen to retain small cells, and it presented retention efficiencies as high as 91 ± 8% for the smallest cells investigated (i.e., *Nannochloropsis*, 3.2 μm) (Table 1). For the slightly larger *Isochrysis* cells (3.8 μm), the retention efficiency dropped significantly down to 69 ± 14%, while the clearance and retention rates decreased more than half compared to *Nannochloropsis*. However, this difference in clearance rates was not enough to reach statistical significance (*p* = 0.08). Being almost six times lower in size, *Phaeodactylum* cells (21.7 μm) showed a further decrease in retention efficiency, which approached 48 ± 13%.

All performance metrics of *A. cannabina* were consistently higher for both the smallest and biggest cells under investigation (i.e., *Nannochloropsis* and *Phaeodactylum*), with the retention efficiencies reaching 41 ± 8 and 46 ± 15%, respectively. Surprisingly, *Isochrysis* was deemed to be the least preferable substrate, as its retention efficiency dropped to half. Considering that *Isochrysis* was the only motile species among those investigated, it is tempting to speculate that cell motility plays a role in the filtration performance of *A. cannabina*.

The species *C. reniformis* exhibited the highest clearance rate and retention efficiency for *Isochrysis* (1.4 ± 0.2 mL h^−1^ g^−1^ and 55 ± 6%, respectively), closely followed by *Nannochloropsis* (1.1 ± 0.2 mL h^−1^ g^−1^ and 46 ± 3%, respectively). A much lower retention efficiency was observed for the large diatom cells (29 ± 10%). In addition, the clearance rate measured for *Phaeodactylum* (0.5 ± 0.3 mL h^−1^ g^−1^) was statistically lower than those for *Nannochloropsis* and *Isochrysis* (*Nannochloropsis* vs. *Phaeodactylum*: *p* = 0.004; *Isochrysis* vs. *Phaeodactylum*: *p* = 0.0006).

With regard to *S. foetidus*, all performance metrics were considerably higher when tested against the larger cells of the pelagic diatom *Phaeodactylum* (Table 1). Indeed, its retention efficiency for *Phaeodactylum* (66 ± 21%) was the highest observed among the examined sponge species, implying that *S. foetidus* might feed more efficiently on big particles. On the contrary, *S. foetidus* demonstrated less preference for cells of smaller size, as the retention efficiency for *Nannochloropsis* (36 ± 13)%) and *Isochrysis* (30 ± 24%) were 46% and 55% lower, respectively. Similarly with *A. cannabina*, it was evident that the filtering activity was not necessarily limited by an increase of particle size. This comes in contrast to Duckworth et al. [33], who examined three tubular sponges (i.e., *Aplysina lacunosa*, *Callyspongia vaginalis* and *Niphates digitalis*) in situ and found that particle retention by all of them decreased as particle size increased from 0.7 to 18 μm.

Overall, the examined sponges exhibited widely disparate retention efficiencies for the different microalgae, which ranged from 24% up to 91% when the initial cell concentration was 5 × 10^5^ cells mL^−1^. This range is much broader than the one reported by Pile and Witman [14], who investigated a single boreal sponge (i.e., *Mycale lingua*) against several types/classes of planktonic cells <10 μm present in the ambient seawater (72–93%), or the range of values obtained in various studies for nano- and pico-plankton (75–99%; [13,16,38,39,40]). On the basis of clearance rates, the best performance in our study was observed for *A. oroides* exposed to *Nannochloropsis* cells (5.4 ± 2.0 mL h^−1^ g^−1^), while the worst one was noticed for *S. foetidus* with *Isochrysis* cells (0.4 ± 0.3 mL h^−1^ g^−1^). In view of the overall feeding efficiency of sponges on phytoplankton (i.e., all three tested microalgae as a total), *A. cannabina* had the lowest performance (37 ± 12%), followed with slightly higher values by *C. reniformis* (43 ± 13%) and *S. foetidus* (44 ± 19%). On top of them, *A. oroides* displayed the highest retention levels, with an overall feeding efficiency of 70 ± 21%. However, the variances in overall retention efficiencies and clearance rates among the different sponges were determined as not significant (*p* = 0.170 and 0.165, respectively) via one-way ANOVA analysis.

What is more noteworthy is the divergence in the cleaning performance of two examined sponges against cells of similar size, but of different microalgal species (i.e., *Isochrysis* and *Nannochloropsis*). Statistical analyses confirmed that this is the case for *A. cannabina* (*p* < 0.001) and *C. reniformis* (*p* = 0.035). In particular, the clearance and retention rates of *A. cannabina* were almost three times higher for *Nannochloropsis* than for *Isochrysis*. This is in sharp contrast to the results reported by Duckworth et al. [33], who investigated the retention efficiency of three coral reef sponges against diverse microbial substrates dwelling in the surrounding waters and concluded that sponges are unselective feeders for a given particle size. On the other hand, a feeding selectivity for specific microbial substrates among others of comparable size was observed by Maldonado et al. [41]. The latter study showed that two similarly sized bacteria, namely *E. coli* (1 μm in length × 0.4 μm in diameter; non-flagellated strain) and *V. anguillarum* (1.1 μm × 0.6 μm; flagellated), were retained by different rates from the marine sponge *Hymeniacidon perlevis*. This distinctive difference was attributed to the capability of sponges to readjust the intake rate of each microorganism in response to other features rather than size alone. It was further argued that the phagocytosis process in choanocyte chambers might be more complicated for flagellated *V. anguillarum* cells, as flagellum beating in choanocytes can make the engulfing more laborious and, thus, less efficient. This theory could explain the higher cleaning efficiency of non-motile, non-flagellated *Nannochloropsis* cells compared to the motile, flagellated *Isochrysis* that was observed in our study. Moreover, differences in the retention rates of similarly sized cells are also likely to occur as a result of choanocytes’ ability to discriminate microorganisms based on the chemical entities present on their exterior surface, as Wehrl et al. [42] have previously shown for bacteria. 

In our study, we showed that a range of marine sponges are able to exhibit special food preferences on different microalgae. This can be of particular importance for aquaculture applications, as the best-performing bioremediators for the development of IMTA systems could be selected and applied by taking into account the characteristics of microalgae present in each particular area.

### 2.3. Effect of Initial Cell Concentration on Cleaning Capacity

**Figure 5:**
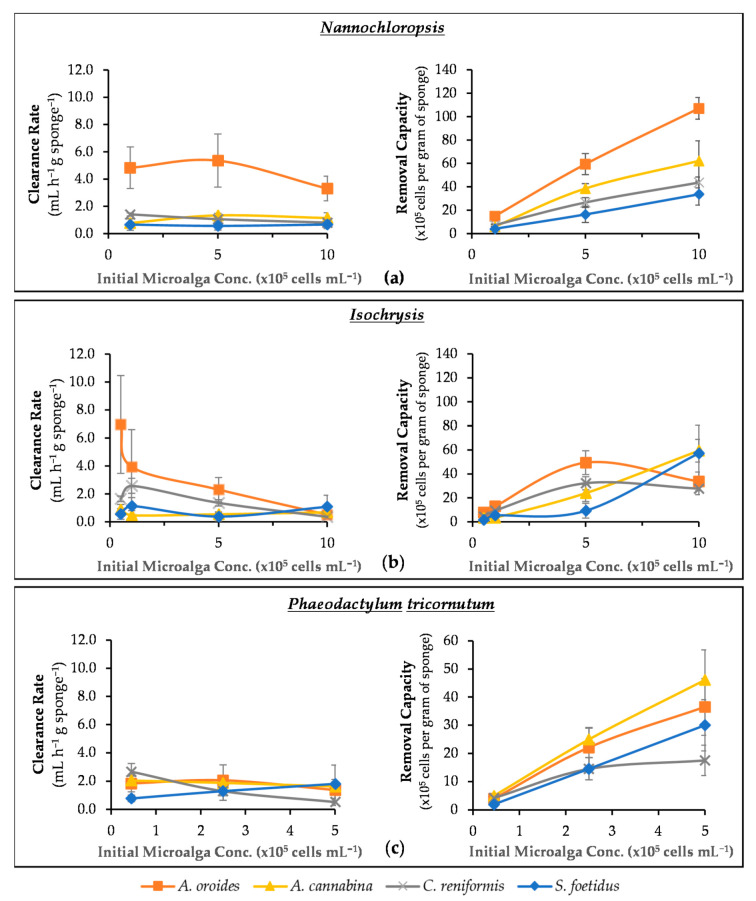
Average cell removal capacity (right) and clearance rate (left) of sponges *A. oroides*, *A. cannabina*, *C. reniformis*, and *S. foetidus* after a 7-h exposure to different initial cell concentrations of microalgae (**a**) *Nannochloropsis* sp.; (**b**) *Isochrysis* sp.; and (**c**) *Phaeodactylum tricornutum*.

In this series of experiments, we examined how the initial abundance of microalgae in the medium affects the cleaning performance of sponges. In particular, Figure 5a–c presents the clearance rate as well as the total removal capacity of the four sponges under investigation in relation to the initial cell concentration of three microalgae species.

When the four examined sponge species were subjected to *Nannochloropsis* cells (Figure 5a), the increase of cell concentration did not have such a dramatic effect in their clean-up capacity. In particular, *S. foetidus*’ activity did not show any systematic variation with cell concentration, and it steadily provided the lowest *c* values among the four sponges (~0.6 ± 0.1 mL h^−1^ g^−1^). A similar behavior was observed for *A. cannabina*, which provided fairly stable *c* values of ~1.1 ± 0.3 mL h^−1^ g^−1^ over the range of cell concentrations tested. The respective values of *A. oroides* presented a slight decrease (~38%) at concentrations higher than 5 × 10^5^ cells mL^−1^, but it remained the best-performing species with a maximum *c* of 4.5 ± 1.1 mL h^−1^ g^−1^. A clearer trend was evident only for *C. reniformis,* which presented a limited but steady decrease of *c* with increasing cell concentration. All the results were further evaluated by one-way ANOVA, and it was revealed that *C. reniformis* was the only sponge species indicating a significant variance in its clearance rate across the different concentrations of *Nannochloropsis* cells (*p* < 0.00001). 

As a result of the relatively stable clearance rates reported above, the removal capacity of all four sponges against *Nannochloropsis* demonstrated an almost linear increase with cell concentration (i.e., the more cells in seawater, the more cells retained by sponges). This finding suggests that all sponges will be able to remove microalgal cells similar to *Nannochloropsis* from aquaculture settings, while their filtration capacity will remain relatively unaffected by temporal or seasonal increases in phytoplankton abundance. Nevertheless, the performance of *A. oroides* looks superior on a quantitative basis, and it would clearly be the best option followed by *A. cannabina*, *C. reniformis*, and *S. foetidus*.

With regard to *Isochrysis* (Figure 5b), the four sponges responded differently to concentration changes. In particular, *A. cannabina* and *S. foetidus* were generally characterized by relatively low clearance rates, which remained fairly stable over the range of cell concentrations tested. As a consequence, the removal capacity exhibited a steady increase with cell concentration for both species, but this trend was more prominent for *A. cannabina*. These two sponges attained the same maximum removal capacity at the highest cell concentration tested (i.e., 1 × 10^6^ cells mL^−1^), and they were able to retain ~5.8 × 10^6^ cells per gram of sponge wet weight within 7 h of exposure. It could therefore be inferred that the specific sponges could respond efficiently even in marine systems with rather high microalgae loadings.

The cleaning effect of *A. oroides* was considerably faster at the lowest cell density tested (5 × 10^4^ cells mL^−1^), providing an average clearance rate of 7.0 ± 3.5 mL h^−1^ g^−1^, but it slowed down sharply at higher cell concentrations. It is worth stressing that one individual fragment of *A. oroides* exhibited the highest *c* value among all four sponges, and it reached 12.5 mL h^−1^ g^−1^ at *Isochrysis* concentration 5 × 10^4^ cells mL^−1^. This was almost five times higher than the *c* of the next best-performing species, *C. reniformis*, which showed a maximum value of 2.6 ± 0.5 mL h^−1^ g^−1^ at a concentration of 10^5^ cells mL^−1^. Regarding removal capacity, the same two sponge species (*A. oroides* and *C. reniformis*) followed a similar parabolic pattern in relation to *Isochrysis* concentration and exhibited maximum retention at 5 × 10^5^ cells mL^−1^ (4.9 ± 1.0 × 10^6^ and 3.2 ± 0.6 × 10^6^ cells retained per gram of *A. oroides* and *C. reniformis*, respectively). This trend suggests that, above a critical concentration of *Isochrysis*, the aquiferous system of the two sponges may gradually become engulfed and saturated by microalgal cells. Similar parabolic patterns were previously observed for the removal capacity of the marine sponge *Hymeniacidon perleve* tested against different concentrations of total organic carbon [34]. Moreover, Osinga et al. [25] observed the same pattern by testing the tropical sponge *Pseudosuberites aff. Andrewsi* against microalga *Dunaliella tertiolecta* (~5−8 μm) at a concentration range of 1 × 10^4^−1.3 × 10^6^ cells mL^−1^. The phenomenon of engulfing was also reported by Scheffers et al. [43], who measured the removal of bacteria by encrusting sponges (Demospongiae and Calcarea). 

Investigating the effect on the cleaning performance of sponges by the concentration of *Phaeodactylum* cells was more complicated due to the relatively large biological variation in the experimental data (Figure 5c). This was particularly evident in the cases of *A. oroides*, *A. cannabina*, and *S. foetidus,* for which it was not possible to identify any statistically significant influence of cell concentration on clearance rates. However, the clearance rate of *C. reniformis* exhibited a steady decline with increasing cell concentration. The same species also demonstrated a distinctly different behavior with regard to the effect of cell concentration on removal capacity. With the exception of *C. reniformis*, which appeared less capable to cope with *Phaeodactylum* concentrations higher than 2.5 × 10^5^ cells mL^−1^, all other sponges presented the characteristic increase of removal capacity with cell concentration. The faster filter-feeder for *Phaeodactylum* was *S. foetidus*, which was able to clean 3.5 mL seawater per gram of sponge wet weight, per hour at the highest initial concentration of 5 × 10^5^ cells mL^−1^.

Grazing and retention rates have been reported to be independent of cell abundance [15,42], but many other studies have come to the opposite conclusion [8,25,34,41,44]. Our results highlighted that both theories can stand for certain sponge species. More specifically, the species *A. cannabina* and *S. foetidus* exhibited a concentration independence regarding their clearance rate (Table 2). On the other hand, the increase of the microalgae concentration had a significant impact on the cleaning performance of the sponges *A. oroides* and *C. reniformis*. However, this was more obvious for *C. reniformis*, as its clearance rates were considerably affected with concentration changes of all tested substrates (i.e., *Nannochloropsis*; *p* < 0.0001, *Isochrysis*; *p* < 0.00001, *Phaeodactylum*; *p* = 0.00002), meaning that the particular sponge species is very susceptible to microalgal concentration changes. For *A. oroides*, this was only the case for *Isochrysis* loadings (*p* = 0.0024). Remarkably, higher concentrations of these small-sized particles (e.g., 10^6^ cells mL^−1^) led to significantly decreased clearance rates that were almost 93% lower than the ones recorded at low concentration (e.g., 5 × 10^4^ cells mL^−1^). As Osinga et al. [25] suggest, it is more likely that the high concentrations of smalls cells are blocking the aquiferous system, leading to a reduction in pumping activity.

### 2.4. Effect of Light Intensity on Cleaning Capacity

An additional series of in vitro cleaning experiments were performed to investigate if light conditions can exert an effect on the cleaning performance of sponges based on a hypothetical scenario of increased feeding activity in darkness, as well as to test whether significant day-night differences should be expected in a real-use scenario in IMTA systems. The “light vs. dark” experiments were performed at a specific cell concentration for each tested microalga. More specifically, a concentration of 5 × 10^5^, 10^5^ and 2.5 × 10^5^ cells mL^−1^ was applied for *Nannochloropsis, Isochrysis,* and *Phaeodactylum*, respectively. The concentration selected for each microalga was the lowest possible to minimize potential stress on sponges during the experiments, whilst providing high signal intensity with the fluorometric method to keep analytical errors at minimum. To the best of our knowledge, this is the first time the effect of light intensity is examined as a factor affecting the feeding intensity of sponges, using microalgae as a reference substrate.

**Figure 6:**
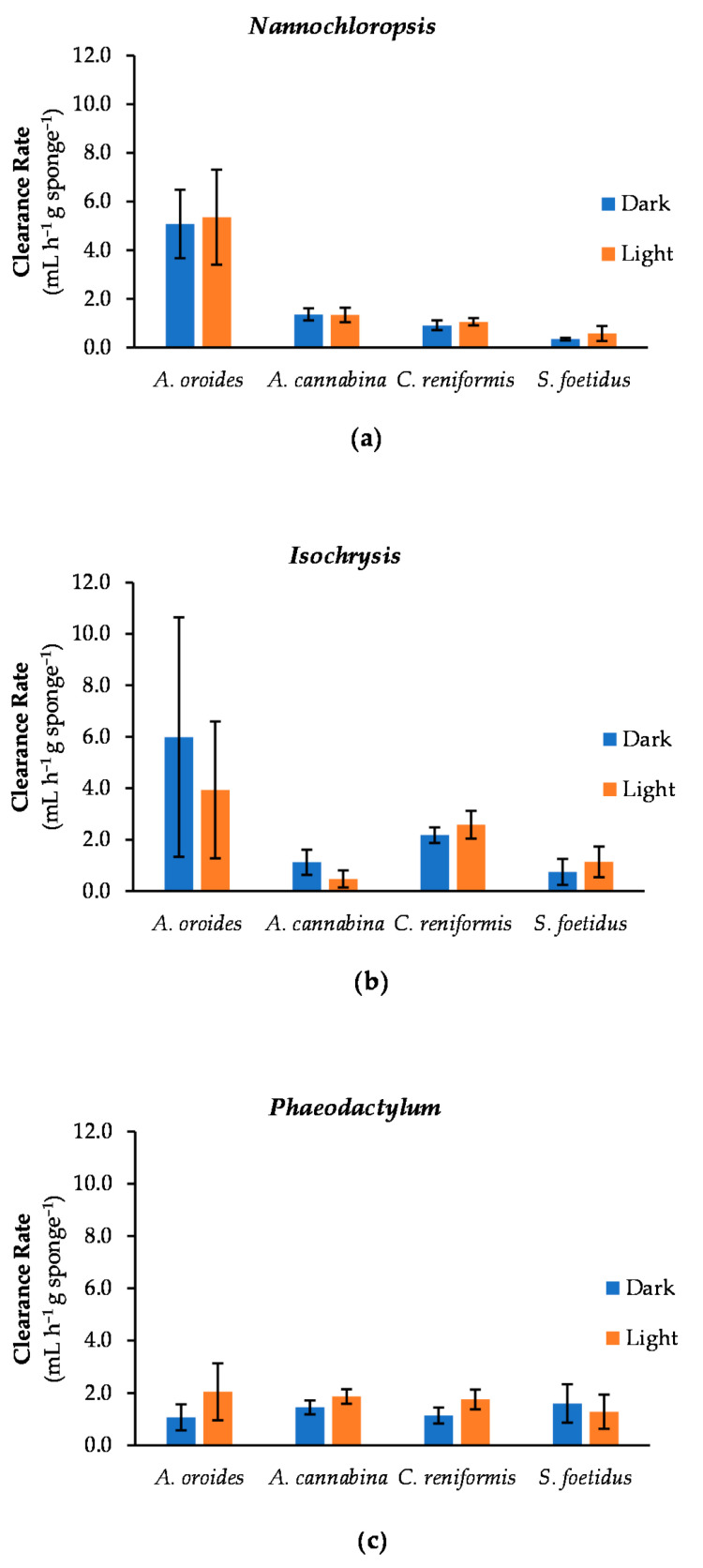
Average clearance rates of sponges *A. oroides*, *A. cannabina*, *C. reniformis*, and *S. foetidus* after a 7-h exposure to the three tested microalgae (**a**) *Nannochloropsis*; (**b**) *Isochrysis*; and (**c**) *Phaeodactylum* under light and dark conditions.

The results for *Nannochloropsis* (Figure 6a) demonstrated no significant variance in clearance rates between light and dark conditions for all examined sponge species (*A. oroides*: *p* = 0.879; *A. cannabina*: *p* = 0.897; *C. reniformis*: *p* = 0.218 and *S. foetidus*: *p* = 0.144). This shows that at least the selected candidates retain a consistent filtering activity regardless of daily light cycles when the seawater is enriched with the particular microalga. Concerning the clearance of *Isochrysis* (Figure 6b)*,* significant differences were observed only in the filtering performance of *A. cannabina*, which tended to be more efficient in darkness (c_dark_ = 1.1 ± 0.5 mL h^−1^ g^−1^) than in light (c_light_ = 0.5 ± 0.3 mL h^−1^ g^−1^) (*p* = 0.0403). Notably, the best performance under darkness was displayed by *A. oroides*, with one of its explants exhibiting both the highest measured clearance activity (*c* = 14.1 mL h^−1^ g^−1^) and retention efficiency (100%) in the present study. However, this extreme case was not sufficient to make a significant difference between light and dark conditions for this species, as the variance in the performance across replicates was particularly high. Lastly, when *Phaeodactylum* is used as a substrate (Figure 6c), significant variances in clearance rates were received for *A. cannabina* and *C. reniformis* (*p* = 0.0407 and 0.0222, respectively), with better performances observed in light (*c* = 1.9 ± 0.3 and 1.8 ± 0.4 mL h^−1^ g^−1^, respectively). However, these values were higher only by 22% for *A. cannabina* and 35% for *C. reniformis* than the ones in darkness.

In general, out of the twelve cases we examined (four sponge species against three microalgae under two different light intensities), only three were associated with significant variances. In particular, the filtering activity of *A. oroides* and *S. foetidus* was not found to be influenced by light conditions. Given that *A. oroides* is a sciaphilic species, which dwells in sheltered places with low light availability (i.e., cave entrances, overhangs, or mesophotic habitats) [45], it is remarkable that it exhibits equally efficient performance under different light regimes for all tested microalgal cells (best performance 12.5 and 14.1 mL h^−1^ g^−1^ in light and darkness, respectively). This suggests an adaptability of the particular sponge to fluctuating irradiance levels. On the other hand, this was rather expected for *S. foetidus*, which is commonly found in shallow habitats exposed to light, but also in darker zones up to 400 m in depth [46]. Nevertheless, this species exhibited the least efficient clearance effects in all experiments (<2.0 mL h^−1^ g^−1^) regardless of light conditions.

Notably, *A. cannabina* can exhibit different filtering performance in the presence and absence of light, depending on the substrate available in the surrounding seawater. Typically, *A. cannabina* can be found in semi-dark habitats (e.g., caves; [47]), but also in places exposed to light (e.g., rocks, stones and calcareous algae at 15–55 m depth; [48]). Thus, we assume the perceived difference in its cleaning performance under dark and light conditions is probably attributed to substrate characteristics. For example, *Isochrysis* are single-celled marine autotrophic microalgae with enhanced cell motility driven by two flagellar systems [49]. Studies have shown that their motility is strongly affected by environmental conditions such as light intensity, pH and nutrients [50]. Recent findings indicate that, during low light periods, *Isochrysis* cells are less motile [51]. Hence, according to Maldonado et al. [41], their capture would require less strain.

Contrastingly, a tendency for increased filtering activity in light rather than in dark conditions was observed for *C. reniformis* and partially for *A. cannabina*, but this difference is significant only when *Phaeodactylum* is used as a substrate. Since *C. reniformis* is inhabiting the littoral zone, with preference on shaded spots [52,53], increased efficiency in the dark would be expected. Our finding may suggest that at least some sponge species can be more active in increased light intensity. Indeed, Reiswig (1971) [11] reported diurnal variances in the pumping rates of the massive, shallow-water marine sponge *Tethya crypta*, with its pumping activity being higher under the regime of light. However, that divergence was attributed to the synchronization of the pumping activity with local water circulation patterns, using the light as a stimulus. Our results are rather inconclusive towards the identification of specific patterns of diurnal differences to filtration rates, suggesting that a more targeted investigation is required to address this concept in future studies. 

## 3. Materials and Methods

### 3.1. Sponge Species Studied

Four Mediterranean sponge species were examined for their filtering activity: (a) *Agelas oroides*, (b) *Axinella cannabina*, (c) *Chondrosia reniformis*, and (d) *Sarcotragus foetidus*. The selection of these species was based on their high abundance (occurrence at high densities) in local natural populations, as well as their massive or erect growth form, since body size has been found to be the major determinant of pumping rates in sponges [54].

*Agelas oroides* Schmidt, 1864 (Agelasida: Agelasidae) is a common massive Mediterranean demosponge, with vivid orange colour and irregular to lobate-digitate shape. Its height varies from 5–25 cm and it can be typically found in 2–40 m water depths, preferably in habitats with low light intensity [45,55]. 

*Axinella cannabina* Esper, 1794 (Axinellida: Axinellidae) is an erect-form sponge, with irregular branches emerging from its body and inner canals 1−3 mm in diameter. It can reach 55 cm in height [56]. It is native in the Mediterranean Sea, with increasing occurrence along its eastern basin [57]. 

*Chondrosia reniformis* Nardo, 1847 (Chondrosiida: Chondorosidae) is acknowledged for its unusual collagenous texture and regenerative properties, which are of biotechnological interest. In contrast to other demosponges, this particular species lacks of skeletal siliceous spicules and spongin fibers [58]. It can generate outgrowths that can extend from the parental body for up to 3 m [59]. It inhabits shaded rocky cliffs or caves at a depth of 1–50 m [52]. 

The sponge *Sarcotragus foetidus* Schmidt, 1862 (Dictyoceratida: Irciniidae) is a medium grey, black or brown demosponge species, which approximates a globular body form. It is quite abundant and one of the largest sponges in Mediterranean coastal ecosystems, typically reaching 1 m in diameter and 50 cm in height with large oscules (0.5–1 cm in diameter) [46].

The selected species represent distinct sponge growth forms (lobate/digitate for *A. oroides*, erect for *A. cannabina*, thickly encrusting for *C. reniformis* and massive for *S. foetidus*). Moreover, they reflect different symbiotic patterns: *A. oroides*, *C. reniformis* and *S. foetidus* are high microbial abundance (HMA) sponges, while *A. cannabina* is a low microbial abundance (LMA) sponge [60]. Finally, some contrasting ecological traits occur among the species. For example, *S. foetidus* is predominantly photophilous [61], while *A. oroides* strictly sciaphilous [45,47,62]. Moreover, *A. cannabina* inhabits deeper water (>20 m depth) [63], while the distribution of *C. reniformis* and *S. foetidus* starts from shallow waters [46,52].

### 3.2. Sponge Sampling

Sponge specimens of the four species were collected in February 2020 from natural populations in two locations in NW Crete, Greece: Stavros (35.588°; 24.075°) for *C. reniformis* and *S. foetidus*, and Souda bay (35.478°; 24.107°) for *A. oroides* and *A. cannabina*. Collection was performed selectively by diving scientists and care was taken to partially collect excess biomass, thus leaving the donor individuals to regenerate. Identification was performed in situ during collection, based on external morphological characters typical for the species. Tissue samples from the candidate species were examined to confirm identification with observation of skeletal features under an optical microscope. Photos of representative individuals of the four studied species at the collection sites are provided in Figure S1.

Collected specimens were transported live in cool boxes with controlled temperature to the Underwater Biotechnological Park (UBPC) of the Hellenic Centre for Marine Research (35.346°; 25.278°), an underwater experimental facility in the open sea. Subsequently, sponges were cut in fragments of approximately 100 g and kept for 3 months in UBPC for regeneration and healing. After the regeneration phase, they were transferred in land-based tanks with continuous flow of filtered NSW (Figure S2) under controlled conditions, resembling local environmental characteristics at the time of transfer (T = 20 °C, pH = 7.6–7.9, Salinity = 39). NSW was passing through a UV disinfection system prior to entering the tank to keep microorganisms at low levels. Before the onset of experiments, the sponges were acclimatized in the tanks for 2 weeks, during which temperature, pH, and salinity were daily monitored to ensure stable conditions. In addition, the wet weight of each sponge explant was measured at the nearest 0.1 g prior and after the conclusion of the experiments to verify that their weight remained consistent throughout the experiments (see Table S1).

### 3.3. Biological Substrates

The lab-based experiments were conducted with the aim to investigate sponges’ filtering activity against three marine microalgae species, each one having different sizes and motility characteristics. One of them was *Nannochloropsis*, which is a green, unicellular, nonmotile microalga belonging to the class Eustigmatophyceae, order Eustigmatales, and family Eustigmataceae. According to Ma et al. [64], the shape of its cell is oval to round, varying in size from 2 to 8 μm, and it has plastids similar to plant cells. In addition, the golden-brown marine flagellated alga *Isochrysis* (class Prymnesiophyceae, order Isochrysidales, family Isochrysidaceae) was selected as being motile, yet having a small size similar to that of *Nannochloropsis* (4–6 µm) [65,66]. It is characterized by fast growth rate, as well as wide temperature and salinity tolerance [67]. The much larger *Phaeodactylum tricornutum* (18–26 μm), a unicellular alga belonging to the class of Bacillariophyceae, order Phaeodactylineae and family Phaeodactylacea, was also included in the experiments. It is a pleiomorphic diatom that can be found in three morphotypes of different size (oval, fusiform or triradiate) depending on the environmental or growth conditions [68]. According to its shape, it can be either slowly motile, or nonmotile. Fusiform cells of a pelagic, nonmotile morphotype were used in our study.

The three aforementioned microalgal species were used as phytoplankton models for simulating extreme eutrophic scenarios and wild cultures of them were provided by the AQUALABS facilities of the Institute of Marine Biology, Biotechnology and Aquaculture (IMBBC) with no prior incubation. For each species, cell size and motility were determined via optical microscopy, as it follows: *Nannochloropsis* sp. (3.2 ± 0.2 μm, non-motile), *Isochrysis* sp. (3.8 ± 0.4 μm, motile), *Phaeodactylum tricornutum* (21.7 ± 1.2 μm, non-motile). 

### 3.4. Experimental Procedures

The protocol for determining the clearance rates of microalgae by marine sponges was based on the methodology proposed by Stabili et al. [9], with minor modifications. More specifically, the lab-scale experiments were performed in 2-L glass jars filled with 1 L of NSW collected from the storage tanks and supplemented with the microalgae of interest. In order to achieve exactly the same cell concentration in all jars, a 26-L suspension of microalgae was initially prepared in a plastic carboy by mixing a specific volume of the original microalgae culture with NSW, followed by gentle stirring. After transferring 1-L aliquots into the jars, a single sponge explant was subsequently immersed in each one of them. For each substrate tested, a total of 20 jars were prepared with explants of the four sponges (five replicates for each sponge species), while another five jars containing NSW and substrate (without sponge) were used as controls.

During an experiment, triplicate samples of 350 μL were collected from each jar at ten different points in time (after 0, 0.25, 0.5, 1, 2, 3, 4, 5, 6 and 7 h) and loaded onto a 96-well opaque microplate for measuring microalgal cell concentration (a total of 750 samples collected in each experiment). The quantitation of microalgae was based on the direct fluorometric detection of cellular chlorophyll *a* using a microplate reader (Infinite F200 Pro; Tecan GmbH, Grödig, Austria) with appropriate optical filters and under high-sensitivity settings (Exc: 435 ± 40 nm, Em: 676 ± 29 nm, settling time: 200 ms, detector gain: 50, temperature: 25 °C, number of flushes: 10) [69]. NSW blank samples were also collected and analysed prior to the initiation of each individual experiment. Although, blank values were close to the detection limit, all measurements of microalgae were subjected to blank subtraction to eliminate the background fluorescence signal of NSW.

A calibration curve correlating fluorescence signal with cell density was prepared for each microalgae species and used for cell quantification in the samples. Throughout the experiments, the liquid inside the jars was gently stirred at regular intervals to eliminate potential biases due to gravitational settling of cells and the localized reduction of cell concentration around the feeding sponge. 

The cleaning capacity of the sponges was examined against a broad range of microalgal concentrations ranging from 0.5 to 10 × 10^5^ cells mL^−1^, which approximate those experienced in highly eutrophic systems [70]. More specifically, the initial concentrations of microalgae were 1, 5, 10 × 10^5^ cells mL^−1^ for *Nannochloropsis*, 0.5, 1, 5 and 10 × 10^5^ cells mL^−1^ for *Isochrysis* and 0.5, 2.5 and 5 × 10^5^ cells mL^−1^ for *Phaeodactylum*.

For each microalgal substrate, we also tested sponges’ filtering activity in the presence and absence of light. This refers to the hypothesis that sponges harboring phototrophic microsymbionts may reduce their pumping rate in the presence of light, while they might shift to increased heterotrophy in darkness. In this case, the experiments were performed in the same way as described above, but the jars were totally covered with aluminum foil. 

The effect of cell size, initial cell concentration and light intensity on the sponges’ cleaning performance was determined by performing one-way ANOVA analysis, to track any differences. The significance level (α) was set to 0.05 (*p* ≤ 0.05).

### 3.5. Preliminary Experiments

To ensure that the filtering capability of sponges under laboratory conditions remained constant from day to day, we conducted a series of preliminary experiments using a single marine microalga (i.e., *Isochrysis*) at a concentration of 10^5^ cells mL^−1^. In particular, we monitored the daily filtering capacity of sponges over five consecutive days and the reproducibility of the results was evaluated. Five explants were used from each sponge species and repetitive experiments were performed following the same procedures as described in the previous section. To check differences in the microalgae retention capacity of sponges between days, a one-way ANOVA was performed on the average removal capacity of the five fragments for each species. The significance level (α) was set to 0.05 (*p* ≤ 0.05).

### 3.6. Data Analysis

Clearance rate (*c*) is a measure that indirectly quantifies the filtering activity of marine suspension feeders [29], including sponges [39]. It represents the volume of water cleared of particles per unit time and sponge weight. The depletion of particle concentration driven by sponges’ filtering activity over time follows the exponential function described by Coughlan [29]:

(1) $$C_{t}=C_{0}\times e^{-c\cdot w\cdot t/V}$$

where *C*_0_ and *C_t_* represent particle concentrations (cells mL^−1^) at time 0 and t, respectively, *V* represents the volume of NSW (i.e., 1000 mL) in the jars, *t* is the time (hours) and *w* is the wet weight of sponge (g). For each experiment, the clearance rate was derived by fitting Equation (1) to cell concentration data, as proposed by Turon et al. [32] and Riisgård et al. [24], and by dividing the resulting constant in the exponent by NSW volume and sponge wet weight. The results from the five biological replicates were averaged to obtain the final *c* value for each sponge species. 

Retention rate (*r*) is another common term, widely used to describe sponges’ filtering activity in the literature [26,32,42]. It is defined as the number of particles retained by the sponge, normalized to sponge wet weight (g) and time (hours). This parameter was calculated according to Wehrl et al. [42], using the following equation:

(2) $$r=\frac{1-\left(10^{\left(y60\right)}\right)}{w}C_{0}V$$

where *y* is the slope of the semi-logarithmic graph *C_t_* versus *t* for the linear time interval, multiplied by 60 to give retention rates per hour. 

In the same context, the retention efficiency (*RE*) can be calculated as the percentage removal of microalgae from seawater at a specific sampling point [9]:

(3) $$\%\;RE=100*\frac{\left(C_{0}-C_{t}\right)}{C_{0}}$$

In our study, we calculated the overall retention efficiency of the sponges by using cell concentration from the last sampling point (*t*_9_ = 7 h).

To offset weight differences between sponge species and replicates, we also used the term *Removal Capacity*, which designates the number of microalgal cells removed within specific time period (i.e., *t*_9_ = 7 h) per unit of sponge wet mass [34]:

(4) $$Removal\;Capacity=\frac{\left(C_{0}-C_{t}\right)}{w}V$$

## 4. Conclusions

In the present study, we were able to compare the filtering capacity and selectivity of four demosponges thriving in the Eastern Mediterranean, namely *A. oroides, A. cannabina, C. reniformis*, and *S. foetidus*, against three representative marine microalgae of different size and motility characteristics. This multiparametric investigation was made possible largely by using a high-throughput, microplate-based method for the fluorometric detection of microalgal cells in a large number of samples. To the best of our knowledge, this is the first time that such a methodological approach is applied in studies of this kind. Moreover, this is the first study to systematically assess filtering activity over a broad range of sponges, microalgal substrates, and different experimental setups.

The examined four sponge species showed distinct preferences regarding the filtering of microalgal substrates of different cell size. While *A. oroides* and, in part, *C. reniformis* followed the expected trend for increased clearance rates with decreasing particle size, this was inverted for *A. cannabina* and *S. foetidus*, which clearly shows that preference to particle size is an innate trait that can show substantial variability and should be further examined without strict adherence to expectations. 

Motility of the particulate substrate is another parameter to be considered when dealing with sponge filtering capacity, since an evident preference for non-motile substrates (*Nannochloropsis*) was observed in our experiments at least for two candidate sponges (*A. oroides* and *A. cannabina*) as compared to motile microalgae of the same size class (*Isochrysis*). This could imply that certain sponge species have optimized their aquiferous system for reduced-mobility (e.g., detritic) substrates, being less effective with microorganisms that are able to escape the inhalant flow. 

Abundance of microalgal substrates in the surrounding medium was not found to play a prominent role to the filtration efficiency of sponges in our experiments, when addressing a wide range of concentrations approximating the gradient from oligotrophic to highly eutrophic systems, simulating the conditions prevailing in the vicinity of fish farms. Taking aside the fact that substrate concentrations were experimentally tested excluding other phenomena commonly associated with eutrophic conditions, such as enrichment in nutrients or presence of pollutants, this finding suggests that sponges retain an optimal filtering capacity along a broad spectrum of microalgal concentrations in the seawater. 

No evidence for a potential effect of the presence or absence of light to the filtering performance of the examined sponges was observed for the studied sponges. This could be expected based on the different preferences of the selected candidates to illumination conditions in their natural habitats. This implies that even sciaphilic species, such as *A. oroides* and *A. cannabina*, can effectively be used in adjacence to fish farms, which are commonly located in the open sea and, thus, exposed to light during the day. It remains to be shown, however, that sciaphilic species can be successfully reared in environments where daylight prevails.

All four candidate sponge species, commonly abundant in Eastern Mediterranean coastal habitats, showed the capacity to feed on microalgal cells. Taking aside variations of performance in the presence of substrates of different size and mobility characteristics, *A. oroides* appears as the most efficient filter-feeder, followed by *S. foetidus*. Hence, both species emerge as interesting candidates for bioremediation applications in IMTA scenarios. However, this evidence regards exclusively microalgae, which are a single component of the spectrum of microorganisms constituting the sponge diet. Similar experiments with viruses, bacteria, protists, and other pico- and nanoplanktonic organisms are still essential. Moreover, the ex situ experimental evidence presented herein should be supplemented by in situ experimental approaches, more closely approximating real-life conditions.

## Figures and Tables

**Table 1:** Wet weight of the sponge specimens used in the experiments and results obtained for the retention rate (*r*), the clearance rates (*c*) derived from Coughlan’s exponential model (together with the coefficient of determination R^2^), the removal capacity, and the retention efficiency of the sponges tested against three different types of marine microalgae. Standard deviations are reported in parentheses.

Microalgae	Cell Size (μm)	Sponge Species	Wet Weight (g)	Retention Rate (×10^5^ cells h^−1^ g Sponge^−1^)	Clearance Rate (mL h^−1^ g Sponge^−1^)	R^2^	Removal Capacity (×10^5^ cells g Sponge^−1^)	Retention Efficiency (%)
*Nannochloropsis*	3.2 (0.2)	*A. oroides*	67.9 (5.4)	25.4 (5.4)	5.4 (2.0)	0.98	59.4 (9.1)	91 (8)
		*A. cannabina*	50.4 (7.6)	9.9 (3.6)	1.3(0.3)	0.96	38.5 (8.2)	41 (8)
		*C. reniformis*	84.2 (12.4)	5.0 (1.2)	1.1 (0.2)	0.99	26.6 (4.2)	46 (3)
		*S. foetidus*	106.5 (26.8)	3.1 (1.2)	0.6 (0.3)	0.97	16.4 (6.7)	36 (13)
*Isochrysis*	3.8 (0.4)	*A. oroides*	67.9 (5.4)	10.4 (3.8)	2.3 (0.9)	0.97	49.4 (10.0)	69 (14)
		*A. cannabina*	50.4 (7.6)	2.5 (0.3)	0.6 (0.1)	0.80	23.9 (7.0)	24 (8)
		*C. reniformis*	84.2 (12.4)	6.2 (1.4)	1.4 (0.2)	1.00	32.2 (5.5)	55(6)
		*S. foetidus*	106.5 (26.8)	1.8 (1.3)	0.4 (0.3)	0.96	9.3 (6.3)	30 (24)
*Phaeodactylum*	21.7 (1.2)	*A. oroides*	67.9 (5.4)	8.3 (4.3)	1.4 (0.6)	0.96	36.5 (10.1)	48 (13)
		*A. cannabina*	50.4 (7.6)	13.7 (4.3)	1.6 (0.5)	0.96	46.1 (10.7)	46 (15)
		*C. reniformis*	84.2 (12.4)	4.5 (1.6)	0.5 (0.3)	0.98	17.5 (5.4)	29 (10)
		*S. foetidus*	106.5 (26.8)	14.3 (11.2)	1.8 (1.3)	0.81	30.0 (9.1)	66 (21)

**Table 2:** *p*-values derived from one-way ANOVA test of sponges’ clearance rates resulted from different microalgal concentrations.

	*Nannochloropsis*	*Isochrysis*	*Phaeodactylum*
*A. oroides*	0.196	0.0024	0.405
*A. cannabina*	0.051	0.106	0.245
*C. reniformis*	0.0001	<0.00001	0.00002
*S. foetidus*	0.893	0.127	0.221

## Data Availability

The data presented in this study are available on request from the corresponding author.

## Supplementary Materials

The following are available online at https://www.mdpi.com/article/10.3390/md20010024/s1, Figure S1: Photos of representative individuals of (a) *Agelas oroides*, (b) *Axinella cannabina*, (c) *Chondrosia reniformis* and (d) *Sarcotragus foetidus* taken from their collection sites. Figure S2: Photos of the regenerated fragments of (a) *Agelas oroides*, (b) *Axinella cannabina*, (c) *Chondrosia reniformis* and (d) *Sarcotragus foetidus* in the land-based tanks., Table S1:Mean wet weight of the used sponge fragments, derived from the measurements prior and after the clean-up experiments. These are presented along with standard deviation and relative standard deviation (%RSD).
