# Supplementary material for: A Multi-Species Investigation of Sponges’ Filtering Activity towards Marine Microalgae
Source: Mar Drugs. 2021 Dec 24;20(1):24. doi: 10.3390/md20010024 (PMC8781895; doi:10.3390/md20010024)
Supplement: Supplementary file 1 [file marinedrugs-20-00024-s001.zip › marinedrugs-1463928-supplementary.pdf]

Supplementary Materials

A Multi-Species Investigation of Sponges' Filtering Activity Towards Marine Microalgae

Despoina Varamogianni-Mamatsi [1,2], Thekla I. Anastasiou [1], Emmanouela Vernadou [1], Nikos Papandroulakis [1], Nicolas Kalogerakis [2], Thanos Dailianis [1,*] and Manolis Mandalakis [1,*]

[1] Institute of Marine Biology, Biotechnology & Aquaculture, Hellenic Centre for Marine Research, 715 00 Heraklion Crete, Greece

[2] School of Chemical and Environmental Engineering, Technical University of Crete, 731 00 Chania, Greece

* Correspondence: M.M. mandalakis@hcmr.gr; Tel.: +30-2810-337855; Fax: +30-2810-337822; T.D. thanosd@hcmr.gr; Tel.: +30-2810-337741; Fax: +30-2810-337822

CONTENTS

Table S1. Mean wet weight of the used sponge fragments, derived from the measurements prior and after the clean-up experiments. These are presented along with standard deviation and relative standard deviation (%RSD).

Figure S1. Photos of representative individuals of (a) *Agelas oroides*, (b) *Axinella cannabina*, (c) *Chondrosia reniformis* and (d) *Sarcotragus foetidus* taken from their collection sites.

Figure S2. Photos of the regenerated fragments of (a) *Agelas oroides*, (b) *Axinella cannabina*, (c) *Chondrosia reniformis* and (d) *Sarcotragus foetidus* in the land-based tanks.

Table S1. Mean wet weight of the used sponge fragments, derived from the measurements prior and after the clean-up experiments. These are presented along with standard deviation and relative standard deviation (%RSD).

	A. oroides					*A. cannabina*				
**Fragment No**	#1	#2	#3	#4	#5	#1	#2	#3	#4	#5
**Average wet weight (g)**	75.2	70.6	74.7	67.7	60.9	43.7	57.8	50.2	54.7	60.3
**Standard deviation (g)**	3.5	1.3	3.1	4.2	1.1	6.8	3.4	4.5	2.5	3.3
**%RSD**	5%	2%	4%	6%	2%	16%	6%	9%	5%	6%

	C. reniformis					*S. foetidus*				
**Fragment No**	#1	#2	#3	#4	#5	#1	#2	#3	#4	#5
**Average wet weight (g)**	82.8	90.5	74.0	103.2	79.9	134.8	100.5	77.2	94.4	139.5
**Standard deviation (g)**	4.7	1.9	5.0	0.4	1.7	0.7	8.1	3.6	0.3	6.6
**%RSD**	6%	2%	7%	0%	2%	1%	8%	5%	0%	5%

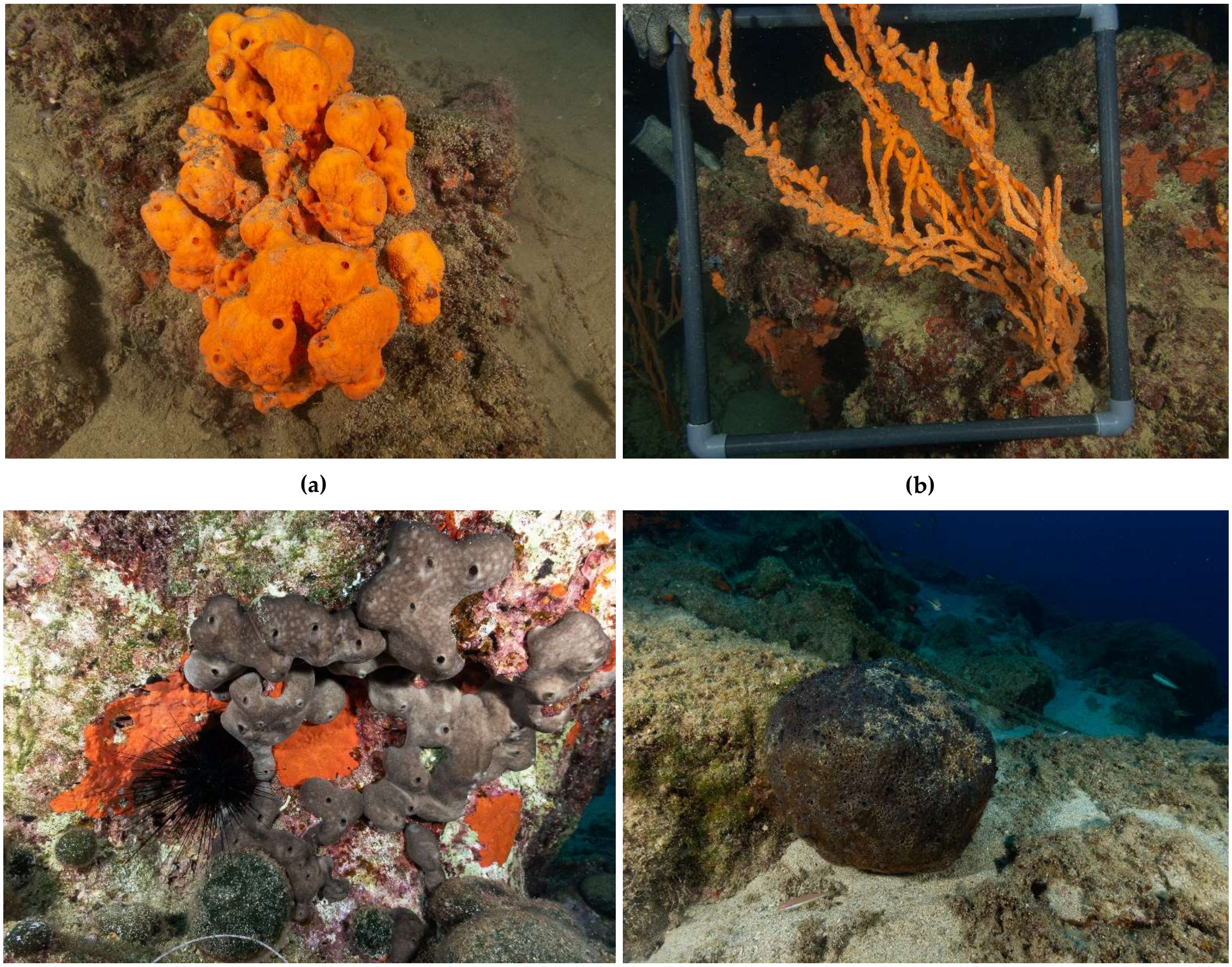

(a) (b) (c) (d)

Figure S1. Photos of representative individuals of (a) *Agelas oroides*, (b) *Axinella cannabina*, (c) *Chondrosia reniformis* and (d) *Sarcotragus foetidus* taken from their collection sites.

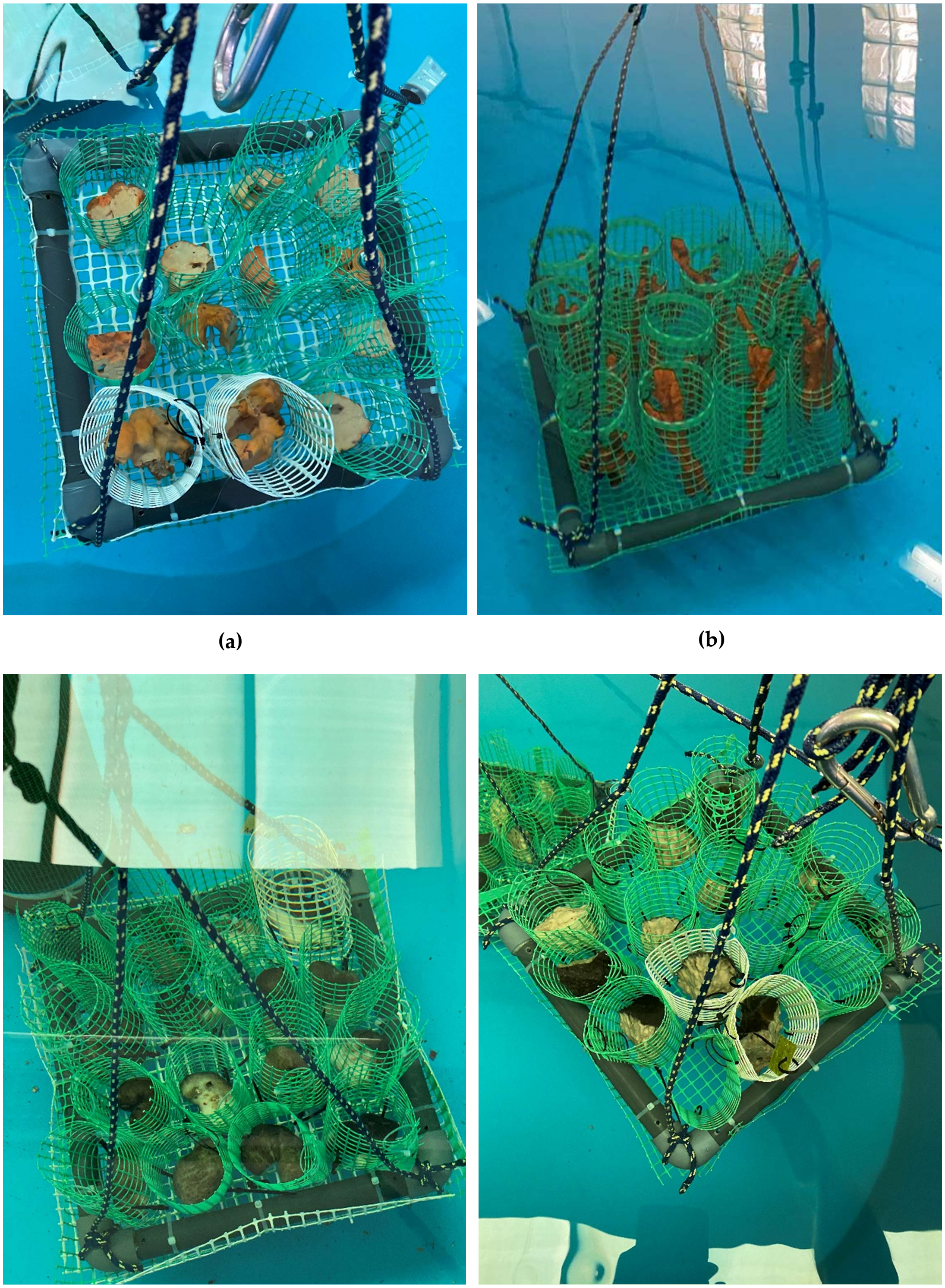

(a) (b) (c) (d)

Figure S2. Photos of the regenerated fragments of (a) *Agelas oroides*, (b) *Axinella cannabina*, (c) *Chondrosia reniformis* and (d) *Sarcotragus foetidus* in the land-based tanks.
